# Nuclear factor-kappaB regulates the transcription of NADPH oxidase 1 in human alveolar epithelial cells

**DOI:** 10.1186/s12890-021-01464-z

**Published:** 2021-03-23

**Authors:** Weijing Wu, Li Li, Xiaoshan Su, Zhixing Zhu, Xiaoping Lin, Jiamin Zhang, Zesen Zhuang, Hongyi Cai, Wenjie Huang

**Affiliations:** 1 Department of Pulmonary and Critical Care Medicine, the Second Affiliated Hospital of Fujian Medical University, Respirology Medicine Centre of Fujian Province, Quanzhou, China; 2 Department of Infectious Disease, General Hospital of Southern Theater Command, PLA, Guangzhou, China; 3 Department of Radiology, Quanzhou First Hospital Affiliated to Fujian Medical University, Quanzhou, China; 4 Department of Medical Imaging, Quanzhou Jinjiang Anhai Hospital, Quanzhou, China; 5 Fujian Medical University, Fuzhou, China; 6 Department of Respiratory Medicine, General Hospital of Southern Theater Command,, PLA, Guangzhou, China

**Keywords:** Acute lung injury, Nuclear factor-kappaB, NADPH oxidase, Tumor necrosis factor-α, NOX1, Reactive oxygen species

## Abstract

**Objective:**

Acute lung injury (ALI) is characterized by inflammation and oxidative stress. Nuclear factor-kappaB (NF-κB) mediates the expression of various inflammation-related genes, including the NADPH oxidase family. This study aimed to identify the potential regulatory role of NF-κB on NADPH oxidases in tumor necrosis factor-α (TNF-α)-induced oxidative stress in human alveolar epithelial cells.

**Methods:**

A549 cells were treated with TNF-α for 24 h to establish ALI cell models. RT-PCR, western blot, assessment of oxidative stress, Alibaba 2.1 online analysis, electrophoretic mobility shift assays and luciferase reporter analysis were employed to identify the potential regulatory role of NF-κB on NADPH oxidases in TNF-α-induced oxidative stress in human alveolar epithelial cells.

**Results:**

The expression of NF-κB/p65 was notably upregulated in TNF-α-stimulated A549 cells. NF-κB knockdown by siRNA significantly inhibited the TNF-α-induced oxidative stress. Moreover, NF-κB/p65 siRNA could inhibit the activation of NOX1, NOX2 and NOX4 mRNA and protein expression in TNF-α-stimulated A549 cells. The next study demonstrated that NF-κB activated the transcription of NOX1 by binding to the -261 to -252 bp (NOX1/κB2, TAAAAATCCC) region of NOX1 promoter in TNF-α-stimulated A549 cells.

**Conclusion:**

Our data demonstrated that NF-κB can aggravate TNF-α-induced ALI by regulating the oxidative stress response and the expression of NOX1, NOX2 and NOX4. Moreover, NF-κB could promote the NOX1 transcriptional activity via binding its promoter in TNF-α-stimulated A549 cells.

**Supplementary Information:**

The online version contains supplementary material available at 10.1186/s12890-021-01464-z.

## Introduction

Acute lung injury (ALI) is a common acute respiratory disease and mainly characterizes by permeability increase, pulmonary edema, as well as impairment of respiratory function [1, 2]. Despite the progressive improvement in the management of critically ill patients and the development of respiratory support therapy, ALI's mortality rate remains as high as 40%. Therefore, there is a need to explore the mechanism and innovative therapeutic strategies of acute lung injury [3].

Numerous studies have found that an imbalance of the antioxidant defense systems and exaggerated production of ROS act as a crucial role in the pathogenesis of ALI [4, 5]. In previous studies, we found that tumor necrosis factor-α (TNF-α) induces excessive inflammation and oxidative stress on alveolar epithelial type II cells (ATII) resulting in cell damage [6]. In the occurrence and progression of ALI, TNF-α is a major mediator of immunity, inflammation, and apoptosis, and has been used to establish the in vitro model of ALI [7–9].

TNF-α is an activator of the nuclear factor-κB (NF-κB) which is essentially important in the mediation of the inflammatory response via regulating the transcription of various pro-inflammatory cytokine, chemokine and adhesion genes [10, 11]. For example, resveratrol represses TNF-α-induced inflammation and ROS generation via the suppression of NF-kB activation [12]. Furthermore, previous research has indicated that the activated NF-κB signaling pathway releases pro-inflammatory cytokines and chemokines such as IL-1β, IL-6 and TNF-α, which are critical to the progression of ALI [13].

Among ROS-generating enzymes, the members of NADPH oxidases (NOXs) enzymes are considered the major source of reactive oxygen species (ROS) in ALI [14–16]. The NOX family (NOX1-5 and DUOX1-2), which are widely distributed in several lung cell types, take part in a wide range of physiological and pathological processes and play critical roles in various pulmonary diseases such as ALI/ARDS, lung infections, obstructive lung disorders and Lung cancer [17, 18]. Accumulating evidence had shown that NADPH oxidases are involved in several diseases by generating excess ROS, particularly Nox1, Nox2 and Nox4 [19, 20]. Previous studies demonstrated that ROS produced by NOX2 participates in TNF-α-induced acute lung injury [21]and NOX2-ROS signaling is involved in the acute inflammatory response via NF-κB activation [11]. In addition, Carnesecchi S et al. [22, 23]has reported that NOX1 is a crucial mediator of hyperoxia-induced ALI and NOX1-generated ROS is a contributor to alveolar cell death.

Increasing evidence suggests that NF-κB could upregulate NADPH oxidases in several cell types [24–26]. However, little is known about whether NF-κB regulation affects NOX family-mediated oxidative stress in acute lung injury. Therefore, we hypothesize that NF-κB might also be a crucial transcription factor for inducing NADPH oxidases expression, involved in the pathogenesis and progression of ALI.

Hence, the present study aimed to investigate the effect and mechanism of NF-κB on TNF-α-induced ATII cells oxidative stress and to better understand the potential regulatory effects of mechanisms on NADPH oxidases expression in ALI.

## Methods

### Cell culture and treatment

Human type II alveolar epithelial cell-derived A549 cells were obtained from Procell life science and Technology Co., Ltd (Wuhan, China) and cultured at Roswell Park Memorial Institute (RPMI)-1640 medium (GIBCO, Los Angeles, CA, USA) with 10% fetal bovine serum (FBS, Gibco), penicillin (100U/ml), and streptomycin (100U/ml). Cells were incubated at 37℃ in a humidified and 5% CO2 atmosphere. A549 cells were plated into 6-well plates with a concentration of 2 × 10^5^ cells/ml and cultured overnight for further experiment. When up to 50%–60% confluence, A549 cells were transfected with specific NF-κB small interfering RNA (50 nmol/L, RiboBio, China) or scrambled control siRNA for 6 h using Lipofectamine 3000 (Invitrogen, USA). After transfection, TNF-α (Pepro Tech, USA) (10 μg/L) was added for another 24 h to establish ALI cell models. The NF-κB/p65 siRNA was designed and synthesized by the RiboBio (Product number: siG09318111121). The NF-κB/p65 gene-specific sequences are as follow: p65-siRNA sense5′-GGACAUAUGAGACCUUCAAdTdT-3′,

antisense 5′UUGAAGGUCUCAUAUGUCCdTdT-3'.

### Assessment of oxidative stress

Oxidative stress was evaluated with reactive oxygen species (ROS) and malondialdehyde (MDA) generation and antioxidant enzymes (total antioxidant capacity, T-AOC; superoxide Dismutase, SOD; total Glutathione S-transferase, TGST). The A549 cells in the inner chamber of each group were harvested and lysed by ultrasonication in the presence of a protease inhibitor. The supernatant was collected for analysis after centrifugation at 14,000 × g for 5 min. The levels of MDA, T-AOC, SOD and TGST in the supernatant were measured using appropriate kits (Beyotime, China) following the manufacturer's instructions.

ROS was measured using the DCFH-DA ROS assay kit (Beyotime, China). Cells were labeled with a 10 µM probe (2,7-dichlorofluorescin-diacetate, DCFH-DA) at 37° C for 30 min in the dark. Then, the cells were washed with PBS and analyzed on an enzyme-labeled instrument by a fluorescence spectrophotometer with an excitation wavelength (488 nm) and an emission wavelength (530 nm).

### RT-PCR

Total RNA from A549 cells was extracted using TRIzol reagent (Invitrogen, USA.) and cDNA was synthesized using Primescript RT Reagent (Takara Bio Inc., Japan). PCR amplification was performed using the SYBR Green PCR kit (Takara Bio Inc., Japan) in a 7500 PCR system (Thermo Fisher Scientific). The relative quantification analysis was calculated using the comparative CT method and β-actin was taken as an endogenous reference. The primers used are presented in Table 1.

**Table 1:** The primer sequences of RT-qPCR (F, forward; R, reverse)

Primer name	Primer sequences
NF-κB/p65	F: 5’-ACAACCCCTTCCAAGTTCCT-3’ R: 5’-TGGTCCCGTGAAATACACCT-3’
NOX1	F: 5’-CTGGGTGGTTAACCACTGGTTT-3’ R: 5’-ACCAATGCCGTGAATCCCTAAG-3’
NOX2	F: 5’-GGAGTTTCAAGATGCGTGGAAACTA-3’ R: 5’-GCCAGACTCAGAGTTGGAGATGCT-3’
NOX3	F: 5’-CCATGGGACGGGTCGGATTGT-3’ R: 5’-GGGGGCAGAGGTAAGGGTGAAGG-3’
NOX4	F: 5’-AGTCAAACAGATGGGATA-3’ R: 5’-TGTCCCATATGAGTTGTT-3’
NOX5	F: 5’-TATGTGACCCTCCATTTGTACCC-3’ R: 5’-CATAACTATTCAAGGATGCTGGC-3’
DUOX1	F: 5’-CTCACCTCTGTTCTTCCTAT-3’ R: 5’-AGAATAAAGAAACCCTGAAG-3’
DUOX2	F: 5’-CCCAAACGTCCATCAACAGA-3’ R: 5’-ATCACCCCAGAAACTCCCCT-3’
β-actin	F: 5’-AGGGAAATCGTGCGTGACATCAAA-3’ R: 5’-ACTCATCGTACTCCTGCTTGCTGA-3’

### Western blot

Total cell proteins were lysed in ice-cold intensified radioimmunoprecipitation assay (RIPA) buffer. Cytosolic and nuclear proteins were prepared according to the manufacturer’s instructions (Thermo, USA), the protein concentration was estimated using BCA protein assay (Thermo, USA). The equivalent amounts of proteins (20–30 μg) were boiled in a sample buffer for 10 min. Proteins were electrophoresed by 10% SDS–polyacrylamide gel electrophoresis and electrically transferred onto PVDF membranes (Millipore, USA). After blocking with 5% skim milk solution, the membranes were incubated overnight at 4 °C with the NF-κB /p65 (1:1000, Abcam, England) NOX1 antibody (1:1000, Abcam, England) respectively. Histone 2A.X (1:1000, signal way, USA) was used as a nuclear marker and β-actin (1:2000, Abcam, USA.) as a cytoplasmic marker. After washing, the membranes were incubated with the secondary antibody (1:5000, Beyotime, China) for 1 h and then visualized and analyzed the enhanced chemiluminescence system. The quantification of the NF-κB /p65, NOX1, NOX2 and NOX4 proteins were analyzed using ImageJ software, with Histone 2A.X or β-actin protein serving as the endogenous reference.

### Electrophoretic Mobility Shift Assay

There are two typical NF-κB binding elements predicted by Alibaba 2.1 online bioinformatic analysis (TRANSFAC) in the proximal promoters (≈1200 bp) of the human NOX1 (GenBank Accession: NG_012567.1 GI:254939587). The double-stranded oligonucleotide probes were designed based on the sequences of these two elements and synthesized by TAKARA (Product number: siG09318111121). The sequences of probes include: probe1 sense (5′- CAGGAAAAAC -3′) and antisense (5′- GTTTTTCCTG -3′), and probe2 sense (5′- TAAAATCCCC -3′) and antisense (5′- GGGGATTTTA -3′). The 3′ end of oligonucleotide EMSA probes were labeled with biotin using Biotin 3′ End DNA Labeling Kit (Thermo, USA). Nuclear protein extracts were obtained with the NE-PER Reagents (Thermo, USA). EMSA was conducted using a Lightshift Chemiluminescent EMSA Kit (Thermo, USA). 1 µl of nuclear extract (10 µg) was incubated in a mixture containing 1 µl of poly dI-dC (1 µg) in a binding buffer with different biotin-labeled DNA probes. For supershift assay, NF-Κb/p65 antibody was added to verify the Specific DNA binding activity of NF-κB protein. After mixture with Loading Buffer, the reaction mixture was run into an 8% nondenaturing polyacrylamide gel to separate from free oligonucleotide, subsequently transferred onto Nylon membranes (Thermo). Biotin-labeled DNA was detected by chemiluminescence, and the membranes were exposed to X-ray film for 2–5 min.

### Plasmids construction, transfection, and dual Luciferase reporter analysis l

The human genomic DNA was used to amplify the human NOX1 proximal promoter fragment (KpnI/ HindIII) (≈1400 bp) using PCR and the deletion mutant one was get using overlap PCR. The fragments were then inserted into the multi cloning sites of the pGL3basic vector (Promega, USA) respectively. The transient transfection of A549 cells was performed using Lipofectamin3000 (Invitrogen, USA) and using Dual-Luciferase Assay Kit (Promega) for the luciferase activity. The luciferase level which stands for the expression of the reporter gene was monitored on an enzyme-labeled instrument (Molecular Devices, USA). The promoter activity was expressed from the relative ratio of firefly luciferase to Renilla Luciferase levels.

### Statistical analysis

Data were shown as means ± standard deviation / mean ± standard error. Statistical significance of the differences among groups was assessed by using a one-way analysis of variance (ANOVA), and then multiple comparisons were performed using the least significant difference (LSD) as a post hoc test. SPSS24.0 was used for all statistical analyses. *P* value < 0.05 was considered to be statistically significant.

## Results

### TNF-α induced NF-κB/p65 activation and translocation to nuclear in A549 cells

**Fig. 1:**
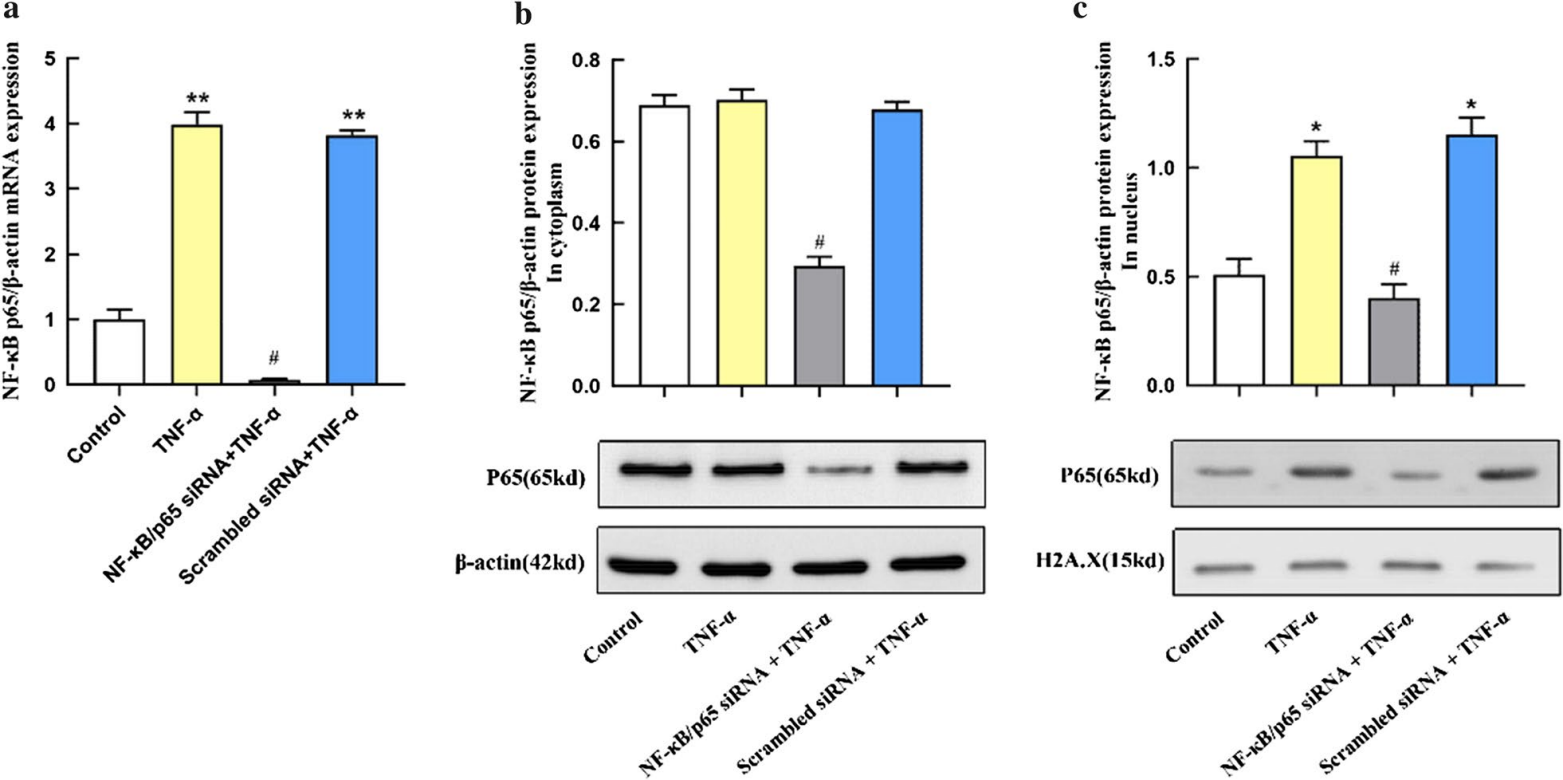
TNF-α induced NF-κB/p65 activation and translocation to nuclear in A549 cells, which were effectively down-regulated by NF-κB/p65 silencing. Control: A549 were cultured without siRNA or TNF-α. TNF-α: A549 were treated with TNF-α (10 ng/ml) 24 h in the absence of NF-κB/p65 siRNA. NF-κB/p65 siRNA + TNF-α: A549 were pre-transfected with NF-κB/p65 siRNA and then treated with TNF-α (10 ng/ml) 24 h. Scrambled siRNA + TNF-α: A549 were pre-transfected with Scrambled siRNA and then treated with TNF-α (10 ng/ml) 24 h. **a** The relative mRNA levels of NF-κB/p65 in the TNF-α-stimulated A549 cells. The values are reported as the Mean ± S.D. (n = 6 for each group). **P < 0.01, compared with Control; #*P* < 0.05, compared with TNF-α and Scrambled siRNA + TNF-α; **b** The relative protein expression of NF-κB/p65 in the cytoplasm in the TNF-α-stimulated A549 cells. Experiments were run in triplicate and repeated 3 times. #*P* < 0.05, compared with TNF-α and Scrambled siRNA + TNF-α; **c** The relative protein expression of NF-κB/p65 in the nucleus in the TNF-α-stimulated A549 cells. Experiments were run in triplicate and repeated 3 times. *P < 0.01, compared with Control; #*P* < 0.05, compared with TNF-α and TNF-α + Scrambled siRNA; The grouping of blots cropped from different parts of the same gel

As NF-κB acts as an essential role in the progression of ALI, we evaluated its expression and activity in A549 during TNF-α induced ALI. We first detected NF-κB/p65 mRNA expression by PCR. The results showed that compared with the control group, the NF-κB/p65 mRNA expression was notably upregulated in A549 treated with TNF-α for 24 h (Fig. 1a). Furthermore, we used western blot to analyze the lever of NF-κB/p65 activation and translocation to nuclear. It was found that TNF-α induced the activation and translocation of NF-κB/p65 (Fig. 1b and c). Besides, the data of RNA interference experiments (the p65 siRNA Group) demonstrated the effective down-regulation of NF-κB/p65 after NF-κB/p65 siRNA transfection.

### Effects of NF-κB/p65 on TNF-α-induced oxidative stress in A549 cells

**Fig. 2:**
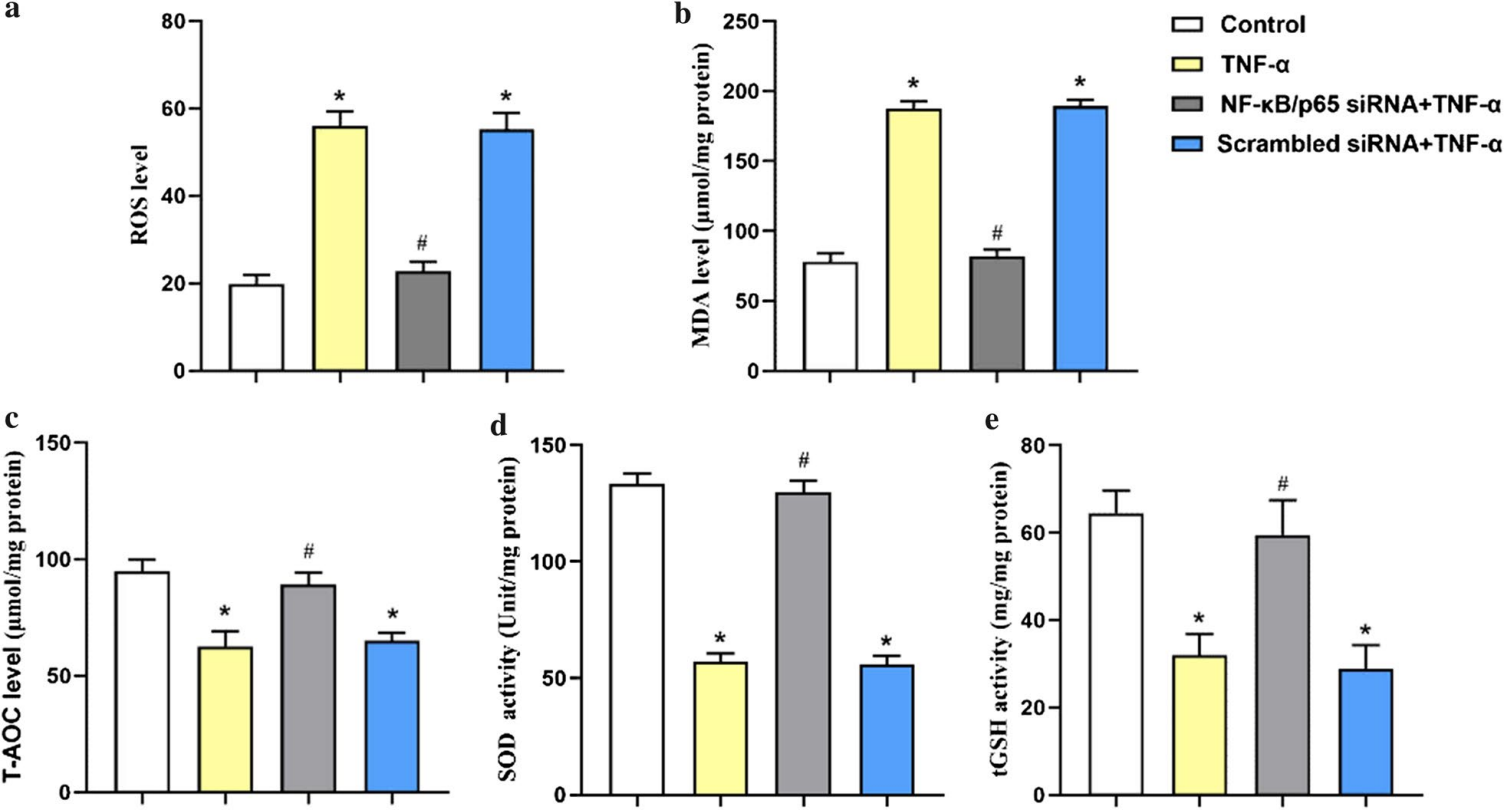
NF-κB/p65 knockdown attenuated oxidative stress induced by TNF-α in A549. Control: A549 were cultured without siRNA or TNF-α. TNF-α: A549 were treated with TNF-α (10 ng/ml) 24 h in the absence of NF-κB/p65 siRNA. NF-κB/p65 siRNA + TNF-α: A549 were pre-transfected with NF-κB/p65 siRNA and then treated with TNF-α (10 ng/ml) 24 h. Scrambled siRNA + TNF-α: A549 were pre-transfected with Scrambled siRNA and then treated with TNF-α (10 ng/ml) 24 h. (A) ROS, (B) MDA, (C)T-AOC, (D) SOD and (E) tGSH. The values are reported as the Mean ± S.D. (n = 6 for each group). *P < 0.05, compared with Control; #*P* < 0.05, compared with TNF-α and Scrambled siRNA + TNF-α

To evaluate the effect of NF-κB/p65 on TNF-α-induced oxidative stress in A549 cells, oxidative stress indices were detected. As shown in Fig. 2, the levels of ROS and MDA were significantly increased while T-AOC, SOD and tGSH were reduced in the TNF-α group compared with the control group. Moreover, we pre-transfected NF-κB/p65 siRNA in A549 cells and the data showed that NF-κB/p65 silencing could notably reduce ROS and MDA levels and increase T-AOC, SOD and tGSH (*p* < 0.05), indicating that NF-κB may attenuate oxidative stress in TNF-a induced ALI (Additional file 2).

### Effects of NF-κB/p65 on the regulation of NOX1, NOX2 and NOX4 mRNA and protein expression in TNF-α-stimulated A549 cells

**Fig. 3:**
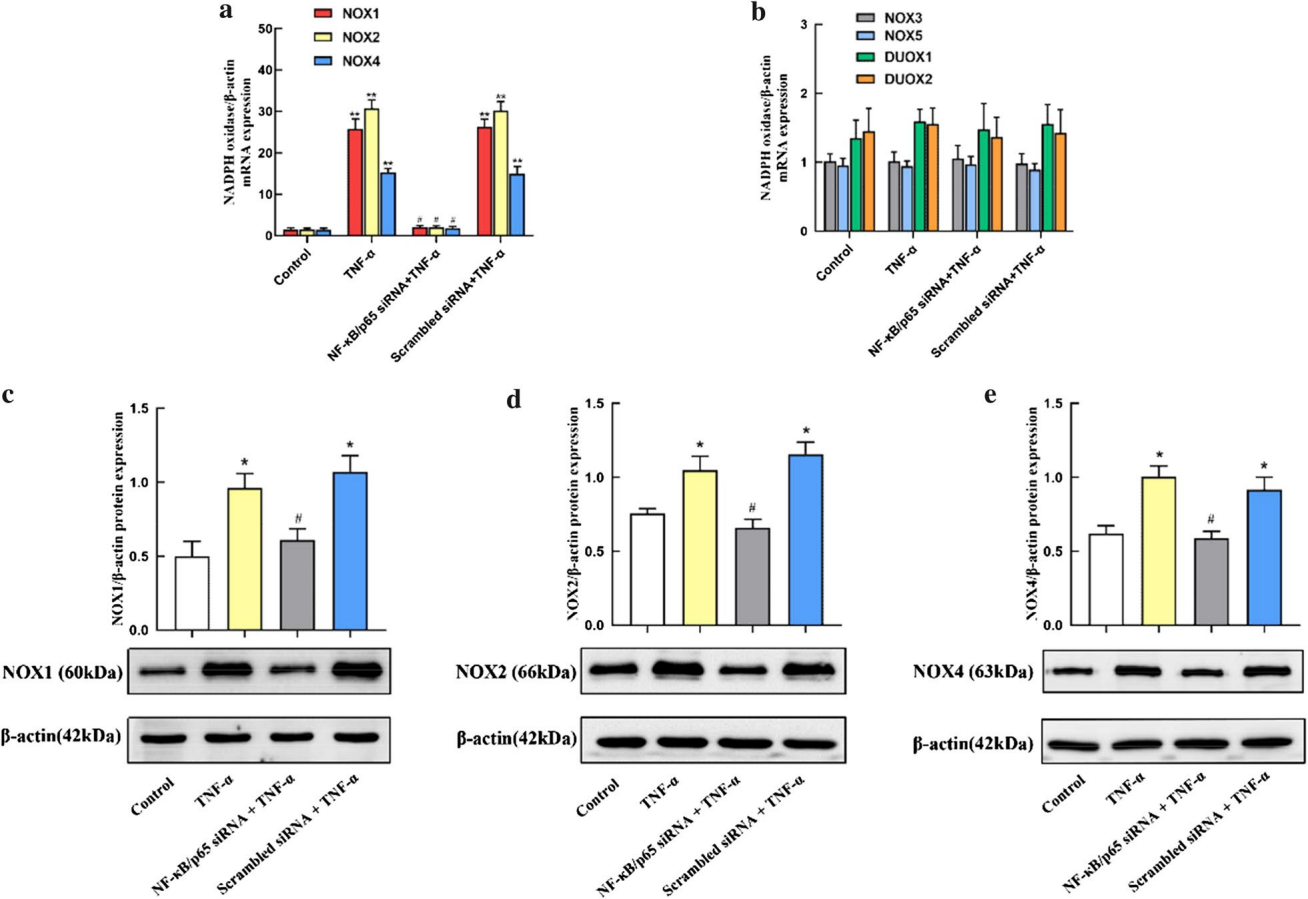
Regulation of NOX1, NOX2 and Nox4 gene and protein expression by NF-κB in TNF-α-stimulated A549 cells. Control: A549 were cultured without siRNA or TNF-α. TNF-α: A549 were treated with TNF-α (10 ng/ml) 24 h in the absence of NF-κB/p65 siRNA. NF-κB/p65 siRNA + TNF-α: A549 were pre-transfected with NF-κB/p65 siRNA and then treated with TNF-α (10 ng/ml) 24 h. Scrambled siRNA + TNF-α: A549 were pre-transfected with Scrambled siRNA and then treated with TNF-α (10 ng/ml) 24 h. **a** The relative mRNA levels of NOX1, NOX2 and NOX4 in the TNF-α-stimulated A549 cells. The values are reported as the Mean ± S.D. (n = 6 for each group). **P < 0.01, compared with Control; #*P* < 0.01, compared with TNF-α and Scrambled siRNA + TNF-α. **b** The relative mRNA levels of NOX3, NOX5, DUOX1 and DUOX2 in the TNF-α-stimulated A549 cells. The values are reported as the Mean ± S.D. (n = 6 for each group). **c**–**e** NF-κB modulated the protein expression of NOX1, NOX2 and NOX4 in TNF-α-stimulated A549 cells. Experiments were run in triplicate and repeated 3 times. *P < 0.05, compared with Control; #*P* < 0.05, compared with TNF-α and Scrambled siRNA + TNF-α. The grouping of blots cropped from different parts of the same gel. The experiments were repeated 3 times

The NADPH oxidases are considered as important originators of reactive oxygen species (ROS) in ALI. Recent studies showed that NF-κB modulates the NOX family gene activity in various cell types. Therefore, we explored whether NF-κB regulates TNF-α-induced NADPH oxidases activation in A549 cells. The mRNA level of NADPH oxidases was analyzed by real-time PCR. Compared with the control group, TNF-α treatment induced NOX1, NOX2 and NOX4 mRNA expression. Moreover, transfection with NF-κB/p65 siRNA markedly diminished the NOX1, NOX2 and NOX4 activation in TNF-α-stimulated A549 (Fig. 3a). However, there was no obvious difference in NOX3, NOX5, DUOX1 and DUOX2 (Fig. 3b).

To certify the role of NF-κB on TNF-α-induced NOX1, NOX2 and NOX4 activation, we detected the protein expression by western blot. As presented in Fig. 3c–e, TNF-α treatment induced NOX1, NOX2 and NOX4 protein activation, and this overexpression in A549 could be inhibited by NF-κB siRNA significantly. In conclusion, these results reflected that TNF-a stimulated NOX1, NOX2 and NOX4 mRNA and protein activation through NF-κB in A549 cells.

### Identification of NF-κB binding sites on the predicted elements of NOX1 promoter

**Fig. 4:**
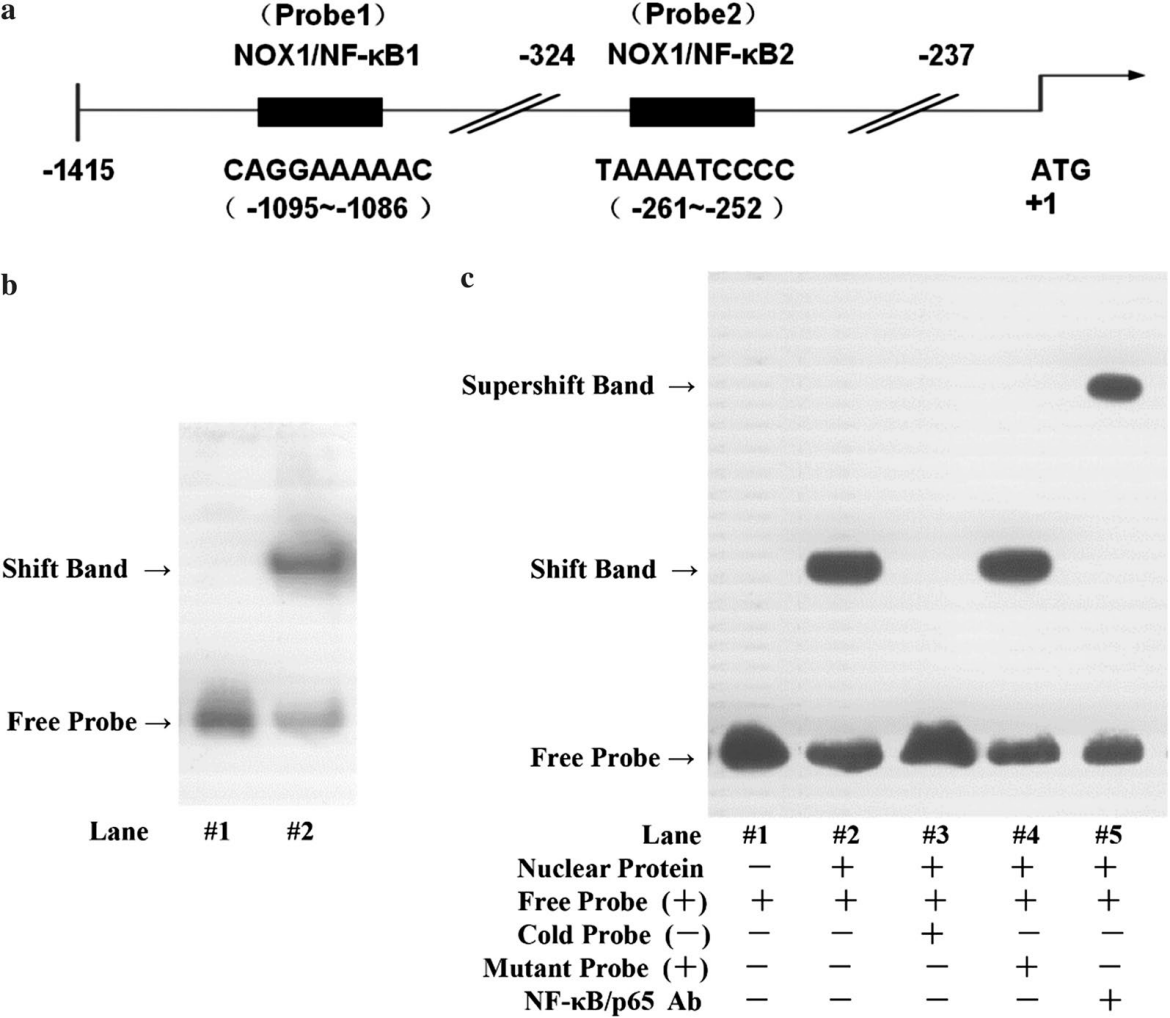
EMSA identification of physical interaction of NF-κB with the predicted binding sites of NOX1 promoter. **a** Schematic drawing of two putative NF-κB binding sites in the proximal promoter of human NOX1 genes based on the bioinformatic analysis. Lane 1: -1095/-1086 bp (NOX1/κB1); Lane 2: -261/-252 bp (NOX1/κB2). **b** EMSA was performed to analyze whether the nuclear protein was able to recognize and bind to the two putative NF-κB binding sites in the promoter of NOX1. Both Lane1 and Lane2 contained the nuclear protein extracted from A549 treated with TNF-α (10 ng/ml) 24 h and different probes. Lane1: probe based on NOX1/κB1; Lane2: probe based on NOX1/κB2. Experiments were run in triplicate and repeated 3 times. **c** EMSA was performed to confirm whether NF‐κB was able to recognize and bind to the putative NOX1/κB2 region of the NOX1 promoter. Nuclear protein was extracted from A549 treated with TNF-α (10 ng/ml) 24 h. Lane 1: contain no nuclear protein; Lane 2: contain both nuclear protein and probe 2; Lane 3: contain both nuclear protein and probe 2, an additional 100-fold molar excess of unlabeled probe 2; Lane 4: contain both nuclear protein and probe 2, an additional 100-fold molar excess of unlabeled probe 2 with scrambled sequences; Lane 5: contain both nuclear protein and probe 2, the nuclear protein was incubated with anti-NF-κB/p65 antibody predictably for 15 min. Experiments were run in triplicate and repeated 3 times

Previous research found that NOX1, but not NOX2 and NOX4, plays an important role in ROS production and cell death in hyperoxia-induced acute alveolar epithelial cell injury [7–9]. Therefore, in this study, we took NOX1 as the target gene of the ALI prevention and control strategy. To further test the regulation of NOX1 expression by NF-κB, bioinformatic analysis was performed on the NOX1 promoter. According to the online prediction website, there might be a strong targeting relationship between NF-κB and NOX1. Two potential NF-κB binding sites in the human NOX1 proximal promoter gene (1439 bp) were assessed using Alibaba 2.1 software (TRANSFACR) (Additional file 1): -1095/-1086 bp (NOX1/κB1), -261/-252 bp (NOX1/κB2). The sequences of two transcription factor binding sites are as below: Nox1/κB1: 5′-CAGGAAAAAC- 3′; Nox1/κB2: 5′-TAAAATCCCC- 3′ (Fig. 4a). To further investigate the functional NF-κB binding sites in NOX1 promoter in TNF-α-stimulated A549, we performed electromobility shift assays (EMSA) (Fig. 4b). The results revealed that a Shift band was detected in lane 2 which containing probe 2 designed based on Nox1/κB 2. To confirm whether NF‐κB was able to recognize and bind to the putative NOX1/κB2 region of NOX1 promoter, a supershift assay was performed. As showed in Fig. 4c, the anti-NF-κB p65 antibody was an addition in Lane 5, resulting in the formation of a bigger complex known as the supershift band, by comparison to the Shift band. Collectively, these findings suggested there is a physical interaction between NF-κB and the putative proximal elements of the NOX1 promoter.

### Regulatory effects of NF-κB on human NOX1 promoter constructs

**Fig. 5:**
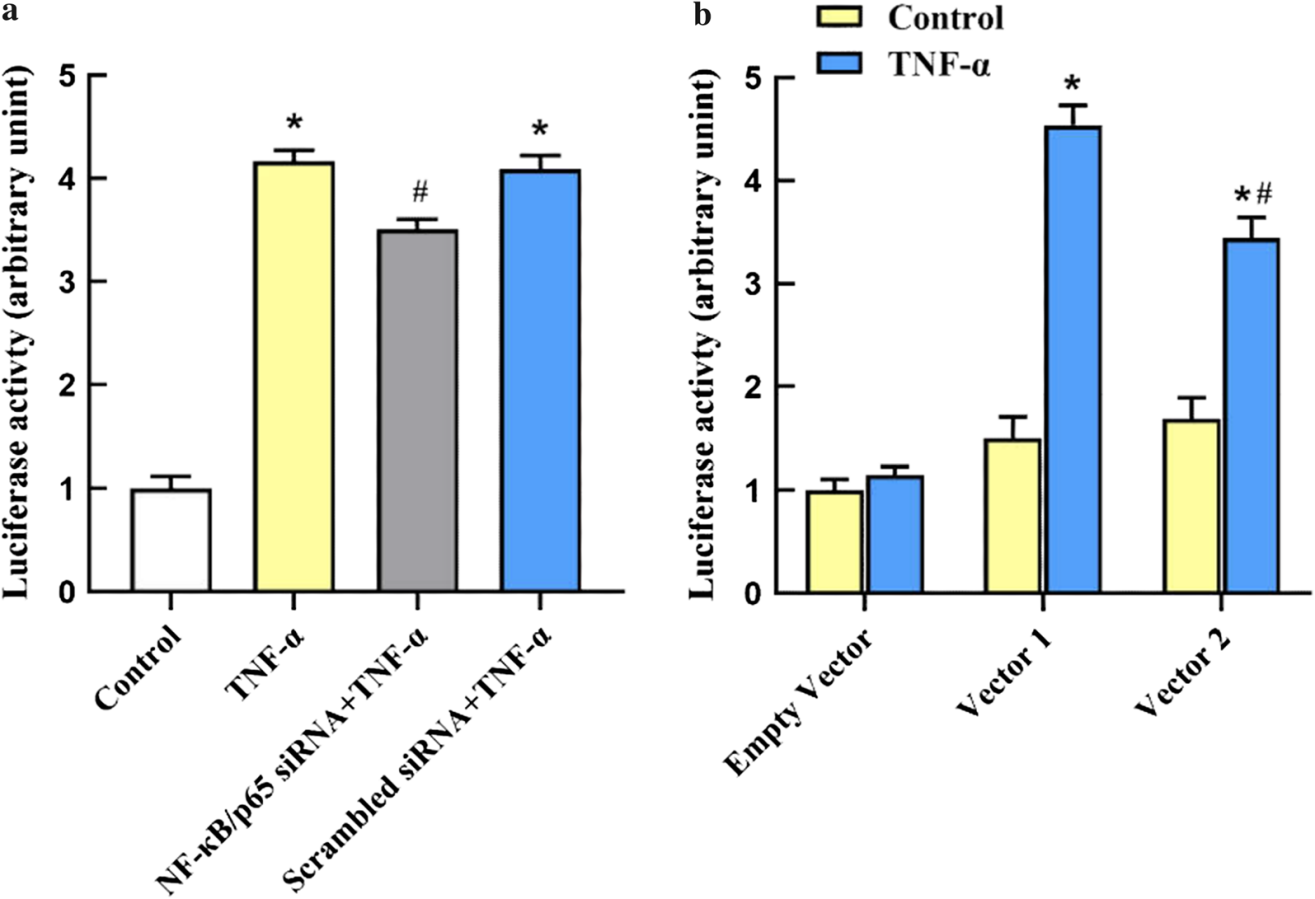
Luciferase reporter analysis of the regulate effects of NF-κB on human NOX1 promoter constructs. **a** The relative luciferase activity of NOX1 promoter in TNF-α-stimulated A549 cells when NF‐κB was knocked down. Control: A549 were cultured without siRNA or TNF-α, but just transfected with Vector1.TNF-α: A549 were pre-transfected with Vector 1 and then treated with TNF-α (10 ng/ml) 24 h in the absence of NF-κB/p65 siRNA. NF-κB/p65 siRNA + TNF-α: A549 were pre-transfected with NF-κB/p65 siRNA and then Vector 1, and treated with TNF-α(10 ng/ml) for an additional 24 h. Scrambled siRNA + TNF-α: compared with the last group, A549 were pre-transfected with Scrambled siRNA instead. Each bar represents the lever of NOX1 promoter activation. The values are reported as the Mean ± S.D. (n = 6 for each group). *P < 0.05, compared with Control. #*P* < 0.05, compared with TNF-α and Scrambled siRNA + TNF-α. **b** The relative luciferase activity of different promoter-reporter plasmids in TNF-α-stimulated A549 cells. Empty vector: A549 were pre-transfected with empty vector, then treated with TNF-α(10 ng/ml) 24 h or not; Vector1: A549 were pre-transfected with Vector1, then treated with TNF-α(10 ng/ml) 24 h or not; Vector2: A549 were pre-transfected with Vector2, then treated with TNF-α(10 ng/ml) 24 h or not. The values are reported as the Mean ± S.D. (n = 6 for each group). *P < 0.05, compared with Empty vector. #*P* < 0.05, compared with Vector1

To further confirm the activation of NF-κB in the NOX1 promoter, a wt-NOX1 promoter-luciferase reporter vector (called Vector1 for short) was constructed and transfected into A549 cells in the absence or presence of NF-κB/p65 siRNA, then treated with TNF-α for 24 h. The results illustrated that TNF-α treatment up-regulated the activity level of NOX1 promoter compared, which could be reduced by NF-κB/p65 siRNA (Fig. 5a). These findings further indicated the involvement of NF-κB in TNF-α-induced activation of the NOX1 promoter.

Next, to investigate whether the putative NF-κB binding sites mediated transcriptional activation of NOX1 genes, we construct a deletion mutant of the human NOX1 promoter-luciferase reporter vector (called Vector2 for short), without the fragment of the NOX1/κB2. Empty vector, Vector1 and Vector2 human NOX1 promoter constructs were transiently transfected into A549 cells, respectively, and the transcription activities were detected in the presence or the absence of TNF-α. As presented in Fig. 5b, TNF-α up-regulated the luciferase activity of Vector1 and Vector2 NOX1 promoters, respectively. Moreover, when the deletion mutant was made in the -261/-252 bp (NOX1/κB2, TAAAAATCCC) region of the NOX1 promoter, the effect of TNF-α on luciferase activity was lower than the integrity of the NOX1 proximal promoters. Collectively, our results suggested that NF-κB could specifically bind to the -261/-252 bp (NOX1/κB2, TAAAAATCCC) region of NOX1 promoter and active NOX1 expression in TNF-α-stimulated A549 cells.

## Discussion

Inflammation and oxidative stress play a crucial role in the pathological processes of Acute lung injury (ALI). In this study, we explored the role and mechanism of NF-κB on TNF-α-induced oxidative stress and the regulatory role of NF-κB on NADPH oxidases in alveolar epithelial type II cells. Our study demonstrated that NF-κB/p65 knockdown inhibited the TNF-α-induced epithelial cells oxidative stress. Moreover, NF-κB/p65 was involved in the regulation of NOX1, NOX2 and NOX4 mRNA and protein expression in TNF-α-stimulated A549 cells. NF-κB activated the transcription of NOX1 by binding to the -261 to -252 bp (NOX1/κB2, TAAAAATCCC) region of NOX1 promoter in TNF-α-stimulated A549. Thus, our findings provide new insights that NF-κB/p65 and NOX1 may act as potential targets and a preventive strategy in the complex regulation of ALI.

As a major mediator of the pro-inflammatory factor, TNF-α was released in the circulation during the early stage of ALI and had been identified in the lung tissues of patients with ALI and ALI mouse models [27]. TNF-α-induced ALI is characterized by cell apoptosis, inflammation response and triggered oxidative stress [7–9]. Thus, we detected the levels of oxidative stress indicators including ROS, MDA, T-AOC, SOD and TGST in different groups. Consistent with previous studies, our research indicated that TNF-α stimulated can induce increased oxidative stress in A549 cells. Therefore, as a nonphagocytic cell, alveolar epithelial type II cells are also the source of TNF-α stimulated oxidative stress, not only the macrophages or neutrophils.

TNF-α has been proved to induce inflammatory and oxidative stress through NF-κB in ALI [9]. NF-κB, a crucial inflammatory inducible transcription factor, mediates the activation of the various pro-inflammatory cytokine, chemokine and adhesion genes. The NF-κB family is present in almost all cells and consists of five members: p65(RelA), RelB, cRel, p50, and p52. In resting cells, the activity of NF-κB is controlled by binding with inhibitory kB (IkB). In response to external stimuli, IκB kinase (IKK) is activated and phosphorylates IκB which is then ubiquitinated and degraded. The NF-κB/p65 then can release from IκB and translocate into the nucleus, where NF-κB/p65 binds to the target gene promoter regions and leads to the transcription [27, 28]. Hence, we explored the NF-κB/p65 expression and the specific molecular mechanism of NF-κB/p65 on TNF-α induced oxidative stress. The data revealed that TNF-α efficiently activated NF-κB/p65 and translocation to nuclear in A549 cells. Furthermore, transfection with NF-κB/p65 siRNA could obviously reduce ROS and MDA levels and increase T-AOC, SOD and tGSH. These findings suggest that NF-κB acts as a crucial role in modulating the regulation of oxidative stress by TNF-α-stimulated alveolar epithelial type II cells.

NADPH oxidases are considered the key source of ROS in ALI. Recent studies showed that increased NADPH oxidases activation in ALI/ARDS mediates the production of reactive oxygen species, the increase of oxidative stress and leading to lung injury [11, 22, 28–30]. Increasing evidence indicates that there is a strong “crosstalk” between NF-κB and NADPH oxidase. There are reported that NF-κB could upregulate NADPH oxidases in several cell types. In epithelial cells of gastric cancer and gastritis, TNF-α stimuli upregulate NOX1 and the inhibition of NF-κB/P65 by shRNA remarkably blocked the upregulation of NOXO1, which is one component forming NOX1 [25]. Moreover, in human colon cancer cell lines, lipopolysaccharide (LPS) treatment increases the expression levels of NOX1 and NOX2 expression, and NF-κB inhibitor attenuates the increase in NOX1 and NOX2 expression [26]. Furthermore, NF-κB is an essential moderator of NOX1 and NOX4 in TNF-α-stimulated human aortic smooth muscle cells [31]. Contrary to these findings, activation of NF-κB has also been proved to be mediated by NOX-derived ROS. In human cardiomyocytes, NADPH oxidase is involved in the regulation of TNF-α-induced NF-κB activation, ROS production and inflammatory [32]. In human pulmonary alveolar epithelial cells, TNF-α-induced activates NF-κB through a NOX2/ROS pathway [33]. Moreover, ROS from NOX2 regulates NF-κB activity in macrophage following Pseudomonas infection [34]. Furthermore, Li et al. [35] reported that NOX2 regulates NF-κB/p65 via ROS in macrophage during *Pseudomonas aeruginosa* infection.

However, whether NF-κB mediates NADPH oxidases activation induced by TNF-α in human lung alveolar epithelial cells has not been completely defined. Our current research showed that TNF-α treatment induced NOX1, NOX2 and NOX4 activation in A549 cells. This observation is in agreement with the prior study, which showed that NOX1, NOX2, and NOX4 expression were significantly increased in LPS-induced ALI [36]. Furthermore, transfection with NF-κB/p65 siRNA markedly diminished the NOX1, NOX2 and NOX4 expression, indicating that the activation of NADPH oxidases induced by TNF-α in ATII cells was mediated by NF-κB. Interestingly, the discrepancies between our studies and previous findings might be due to the difference in cell types and/or the treatment conditions. On the other hand, these data suggest that there may be a regulatory feedback loop between NF-κB and NADPH oxidases that coordinate the inflammatory response and oxidative stress.

NF-κB, the transcription factor, is primarily involved in regulating inflammatory responses by binding to various pro-inflammatory cytokine promoter regions following the treatment with various stimuli, such as TNF and LPS [32, 37]. Hence, there is a possible mechanism that NF-κB regulates NADPH oxidases activation by binding to NADPH oxidases promoter regions in ALI. Previous research found that NOX1, but not NOX2 and NOX4, plays an important role in ROS generation and cell death in hyperoxia-induced acute alveolar epithelial cell injury [22, 23]. We further paid close attention to NOX1 in the current study. Recently, Echizen et al. [25] reported that NF-κB activated the NOX1 gene promoter induced by TNF-α in gastric cancer cells. Therefore, it is of great interest to explore whether the NF-κB regulates the transcription of NOX1 in TNF-α-induced ALI. In our study, Alibaba 2.1 online bioinformatic analysis predicted that the NOX1 promoter-proximal region contained two typical NF-κB binding sites. EMSA revealed that there is a physical interaction between NF-κB and the putative -261 to -252 bp (NOX1/κB2, TAAAAATCCC) region of NOX1 promoter in TNF-α-induced A549 cells. Luciferase reporter assay demonstrated that NF-κB/p65 siRNA could reduce the elevated NOX1 promoter activity stimulated by TNF-α. However, NF-κB/p65 siRNA did not drastically reduce NOX1 promoter activity compared to its ability to reduce NOX1 protein level (Fig. 5a). This difference might be due to the post-translational modification of NOX1 protein, which requires further study. Furthermore, luciferase reporter assay confirmed that NF-κB binding site in the “Nox1/κB2” region of NOX1 promoter was required for the transcription of NOX1, suggesting that NF-κB acts as a crucial role in the transcription of NOX1.

Our study has some limitations that require attention. First, A549 cell lines were used in this study to establish the in vitro model of ALI. Studies have shown A549 is a suitable model for ATII cells because the long-term culture of A549 cells promotes a more ATII-like phenotype [38]. However, A549 is a human lung adenocarcinoma cell line that expresses different levels of oxidative stress compared to normal human ATII cells. Also, we did not investigate the roles of NF-κB and NADPH oxidases in vitro. Therefore, animal experiments are required in future studies to verify these findings.

## Conclusion

**Fig. 6:**
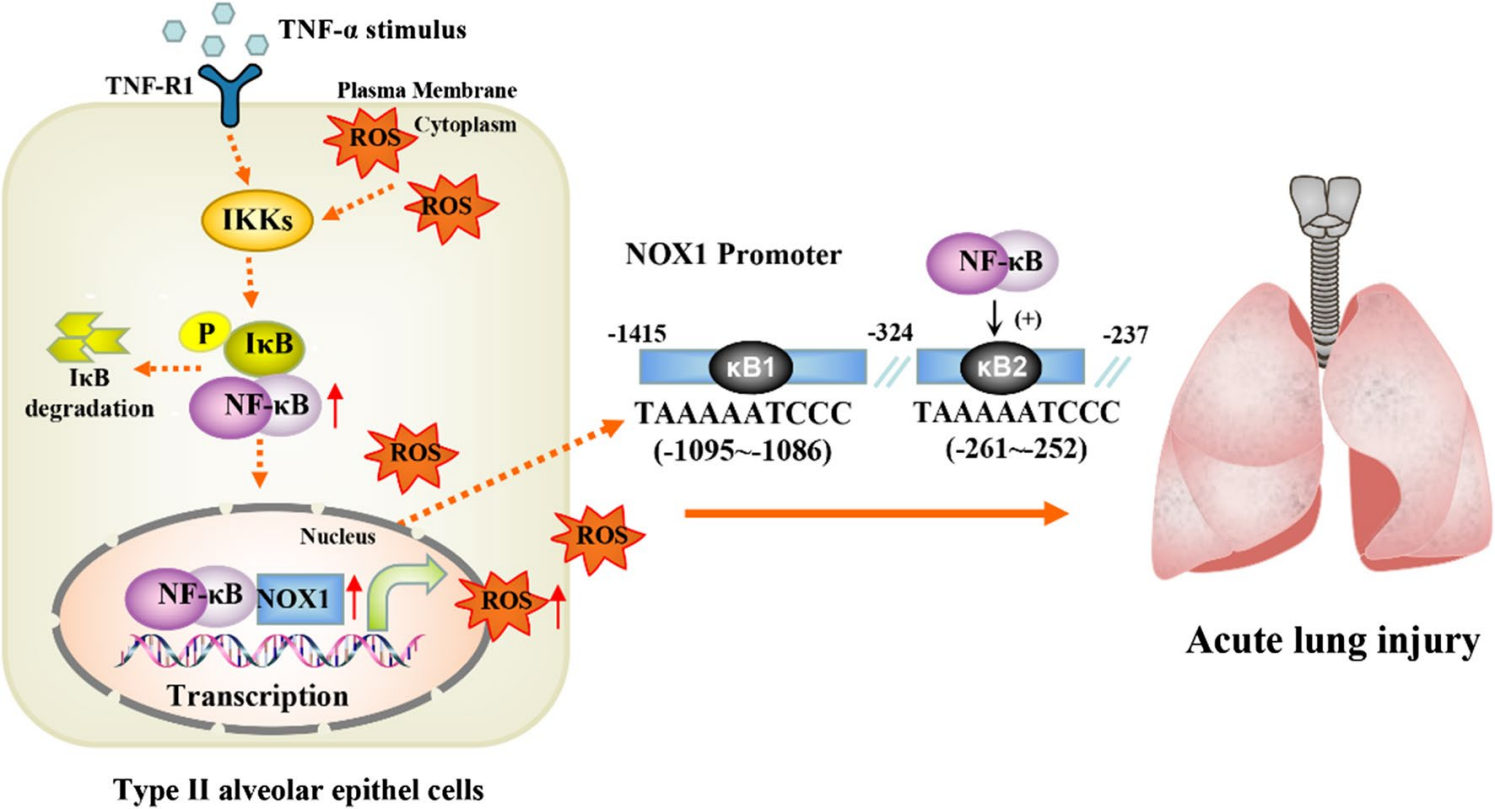
Schematic representation of the mechanism of NF-κB on TNF-α-induced pulmonary inflammatory response via regulating NOX1 in alveolar epithelial type II cells. In TNFα-induced ALI, activated NF-κB can translocate to the nucleus, where it binds to NOX1 promoter regions (κB2, TAAAAATCCC, -261 ~ -252)) and activate the transcription of NOX1 and oxidative stress, which can promote inflammatory and oxidative stress

In the present study, we found that NF-κB can aggravate TNF-α-induced ALI by regulating oxidative stress and the expression of NOX1, NOX2 and NOX4. Our data suggested that NF-κB can activate the transcription of NOX1 by binding to the -261 to -252 bp (NOX1/κB2) region of NOX1 promoter in TNF-α-stimulated A549 (Fig. 6). Our results provide a novel theoretical basis for future research on the transcriptional mechanism of NOX1. Therefore, targeting NF-κB or NOX1 signaling could be a potential strategy for further prevention and treatment of ALI. Further study will verify the NF-κB/NOX1/ROS axis through in TNF-α-induced ALI mouse models.

## Supplementary Information

**Additional file 1.** Two potential NF-κB binding sites in the human NOX1 proximal promoter gene.

**Additional file 2.** **Supplementary Table 1.** The concentration of ROS and MDA of different groups. **Supplementary Table 2.** The concentration of TAOS, SOD and TGSH of different groups.

## Data Availability

All the related data are presented in the manuscript.
