# Supplementary material for: Nuclear factor-kappaB regulates the transcription of NADPH oxidase 1 in human alveolar epithelial cells
Source: BMC Pulm Med. 2021 Mar 23;21:98. doi: 10.1186/s12890-021-01464-z (PMC7988993; doi:10.1186/s12890-021-01464-z)
Supplement: Supplementary file 1 — Additional file 1. Two potential NF-κB binding sites in the human NOX1 proximal promoter gene. [file 12890_2021_1464_MOESM1_ESM.docx]

**Additional file 1:** Two potential NF-κB binding sites in the human NOX1 proximal promoter gene (1439 bp) were assessed using Alibaba 2.1 software (TRANSFACR) (supplementary materials): -1095/-1086 bp (NOX1/κB1), -261/-252 bp (NOX1/κB2) (Fig.4A). Specific results were as follows：

-1544 agttttccac acttacttga attataaaaa caaaaaagtc tggaaaatac caagggttgg

-1484 taagcatgta gaggaagtag aactctcatt cataactctc tgtagtatac atttaggtgg

-1424 tcacttcgga acgggtttgg aattacacag caaagtagaa tatgtgcaaa tctcaggacc

-1364 ctggaatttt actcctgggt atatacctta gagaaactgt agcatatgtg tgacatttga

-1304 tcaacattgt tccatcatca tatccatcag tagtaggatg aatgaataca ttaatgtata

-1244 ttcattcatg caatggcata ttagatagca gtgtaagtga accgcaatta catgtacatg

-1184 tatgaatctc aaaaacccaa tgttgaaaga agcaaaccac agaagcatac atacacactg

-1124 ccaggtttca tttacaaaaa gttcaaaaa **CAGGAAAAAC** taaacaatata ttgcttaggg

**NOX1/κB 1**

-1064 atgcaattat agttagtaaa aatataaaga aaaataacag aatgattacc ccaaatttca

-1004 ggatagtgat tacatccggt ggggtagagg aggggaagaa gatagatgtg atcagggagg

-944 gaaatacaaa gagctttaag atactggaga aaaatagtct attttcttta atctgagtag

-884 tgaacacata gatacttatt ccttaaaatt attctttaag ttacatatgt atgttttata

-824 tactcttctg tgtatatttc accattttag aaaagggaaa aaaaatcagt gcccagagct

-764 gaacacacaa ctctagtaaa tctatcatac tagaagacaa tcatctccat tcttttgagt

-704 gctctgcctc tgtttatttt gaaccaaagt gcacttttat acttgttaaa ttttctcttg

-644 ctctatttgg cccttctttt cacttgtcct tccagccagt caagttctcc ccaaagccat

-584 catcatatat gtcaaccaca gatcatcctc caggggaact ggtatgctaa agtttctgag

-524 ctagccaggc tgaaatccaa atggcagccg gcagatgtgg caacagtttg aaaagtgcac

-464 tttgaaacag cttccttacc acacacgctt ccctccctac ttctcctgaa gtaatctgtt

-404 tacagaccca gactaataat cttttttatg agaaacttta gcaaatcttt tatctaggaa

-344 ggcaatgctt cacattaggt catgttgata agatgatgag agagaatatt ttcatccaag

-284 aatgttgcta tttcctgaag cag **TAAAATCCCC** acaggta aaacccttgt ggttctcata

**NOX1/κB 2**

-224 gatagggctg gtctatctaa gctgatagca cagttctgtc cagagaagga aggcagaata

-164 aacttattca ttcccaggaa ctcttggggt aggtgtgtgt ttttcacatc ttaaaggctc

-104 acagaccctg cgctggacaa atgttccatt cctgaaggac ctctccagaa tccggattgc

-44 tgaatcttcc ctgttgccta gaagggctcc aaaccacctc ttgac**ATG**
