# Supplementary material for: Nuclear factor-kappaB regulates the transcription of NADPH oxidase 1 in human alveolar epithelial cells
Source: BMC Pulm Med. 2021 Mar 23;21:98. doi: 10.1186/s12890-021-01464-z (PMC7988993; doi:10.1186/s12890-021-01464-z)

Fig.1 **B** The relative protein expression of NF-κB/p65 in cytoplasm in the TNF-α-stimulated A549 cells

NF-κB/p65


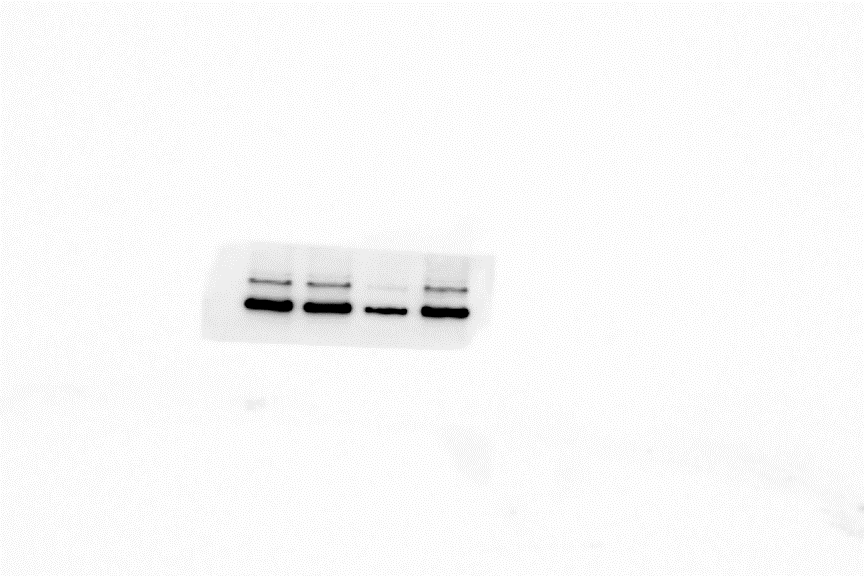


β-actin (The left gel)


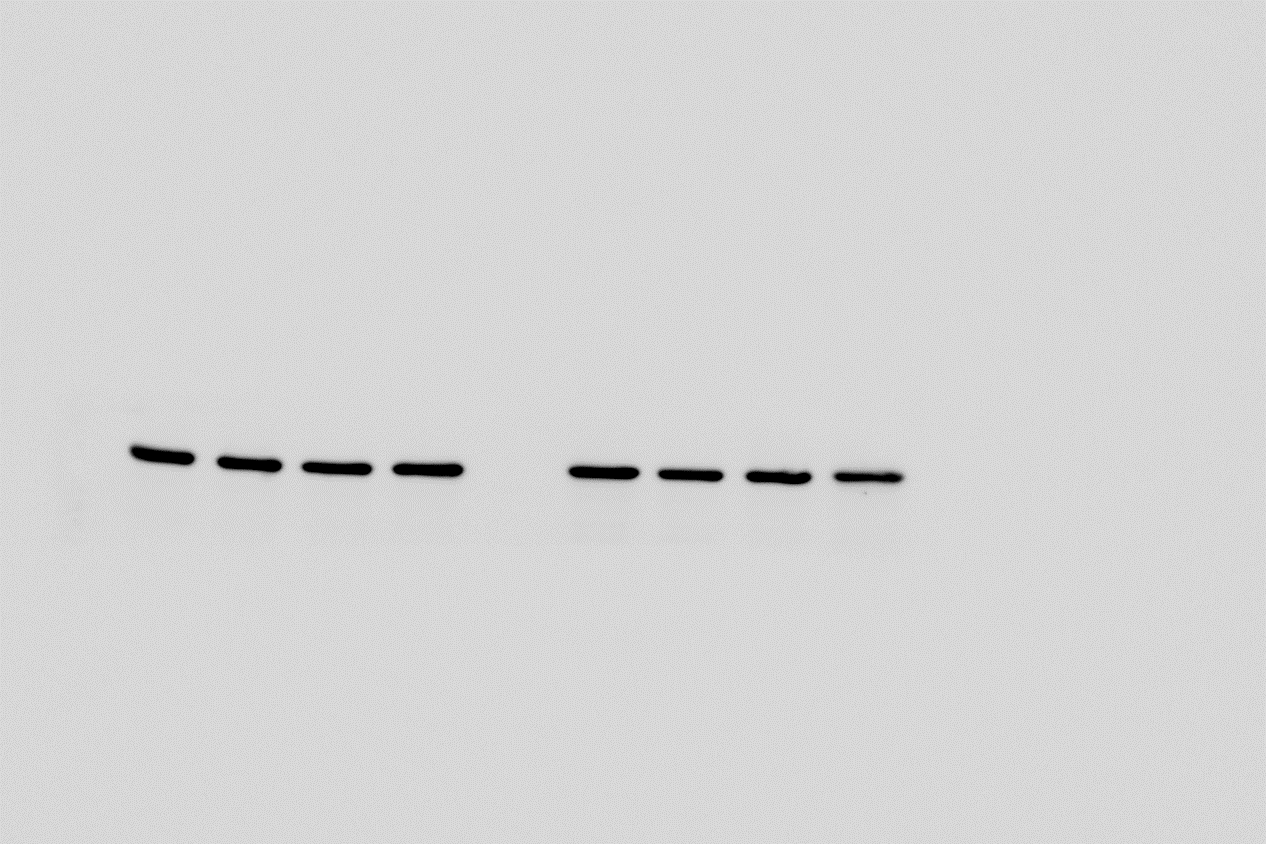


C. The relative protein expression of NF-κB/p65 in nucleus in the TNF-α-stimulated A549 cells.

NF-κB/p65


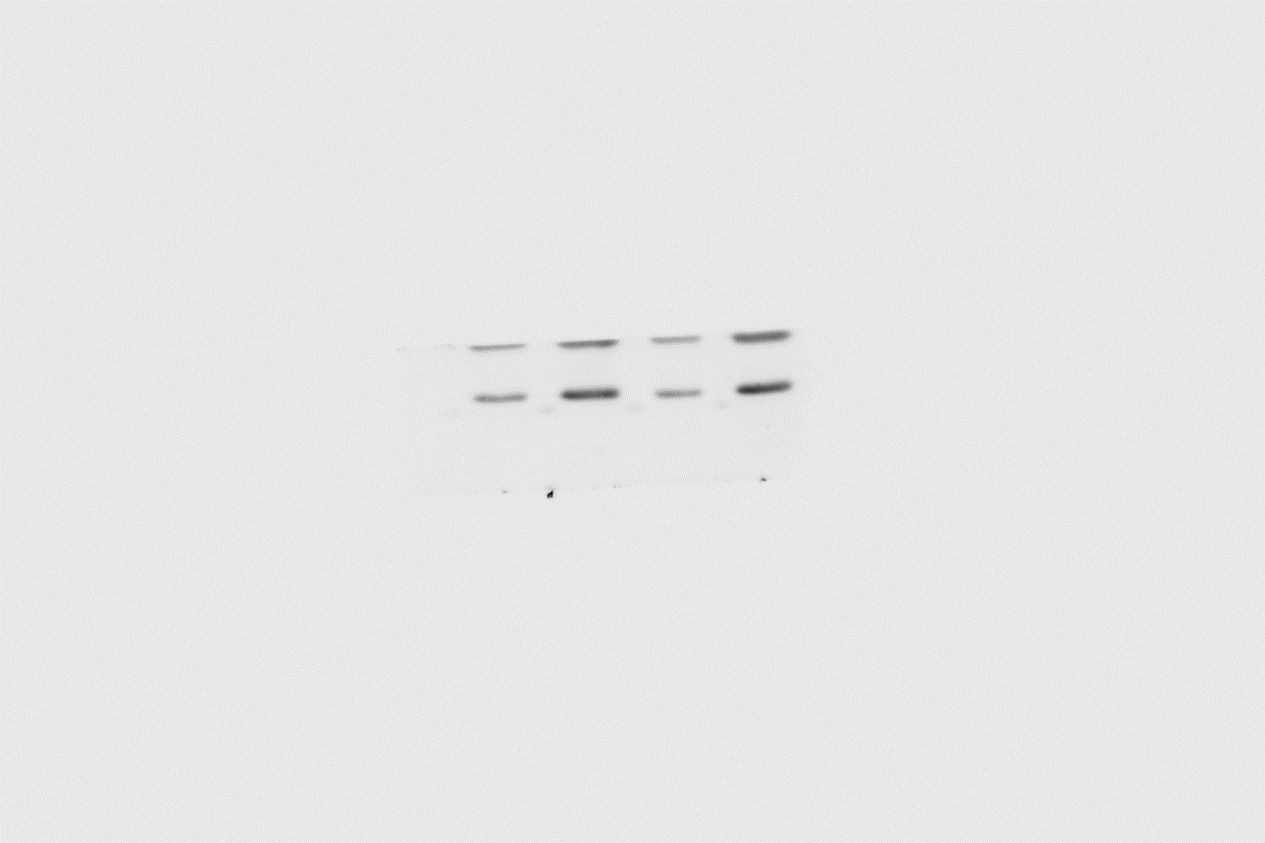


H2A.X


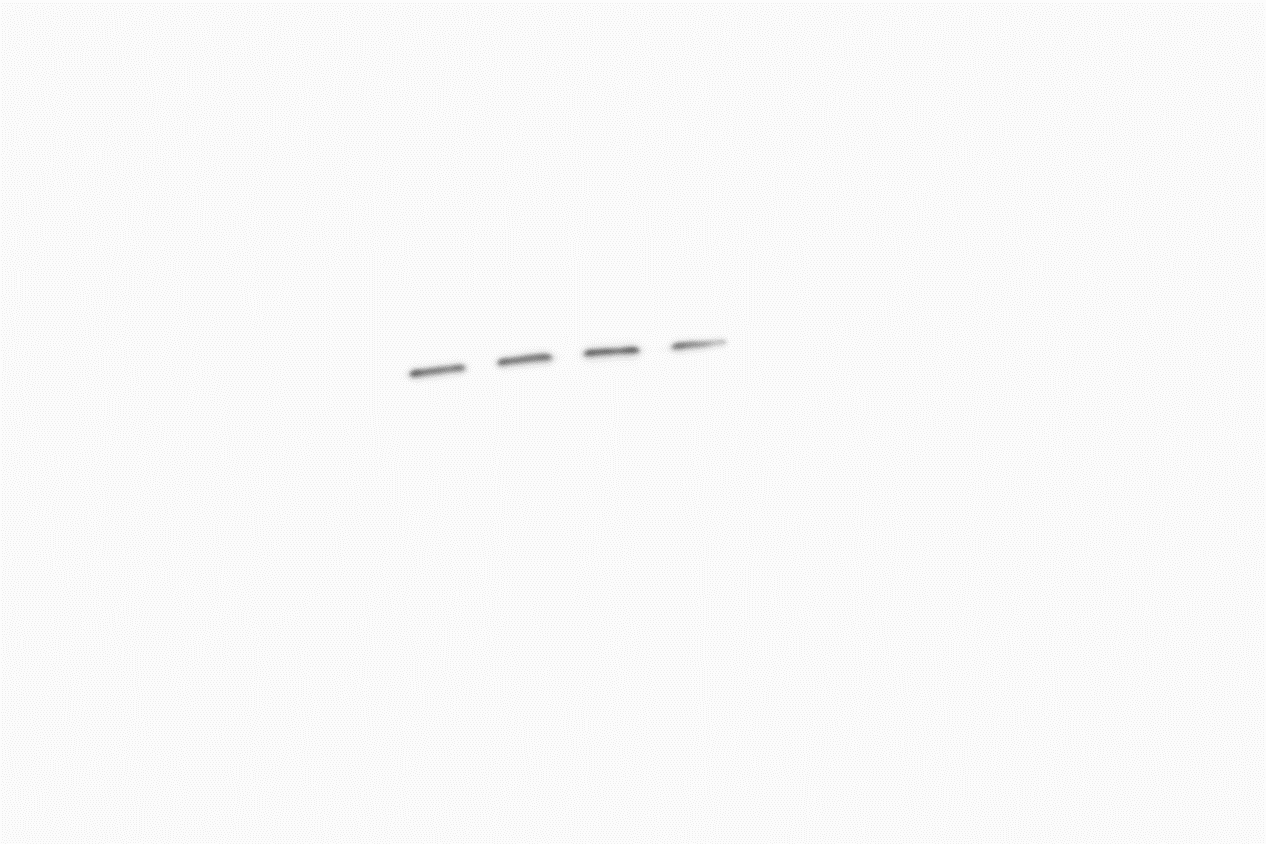


C. NF-κB modulated the protein expression of NOX1in TNF-α-stimulated A549 cells.

NOX1


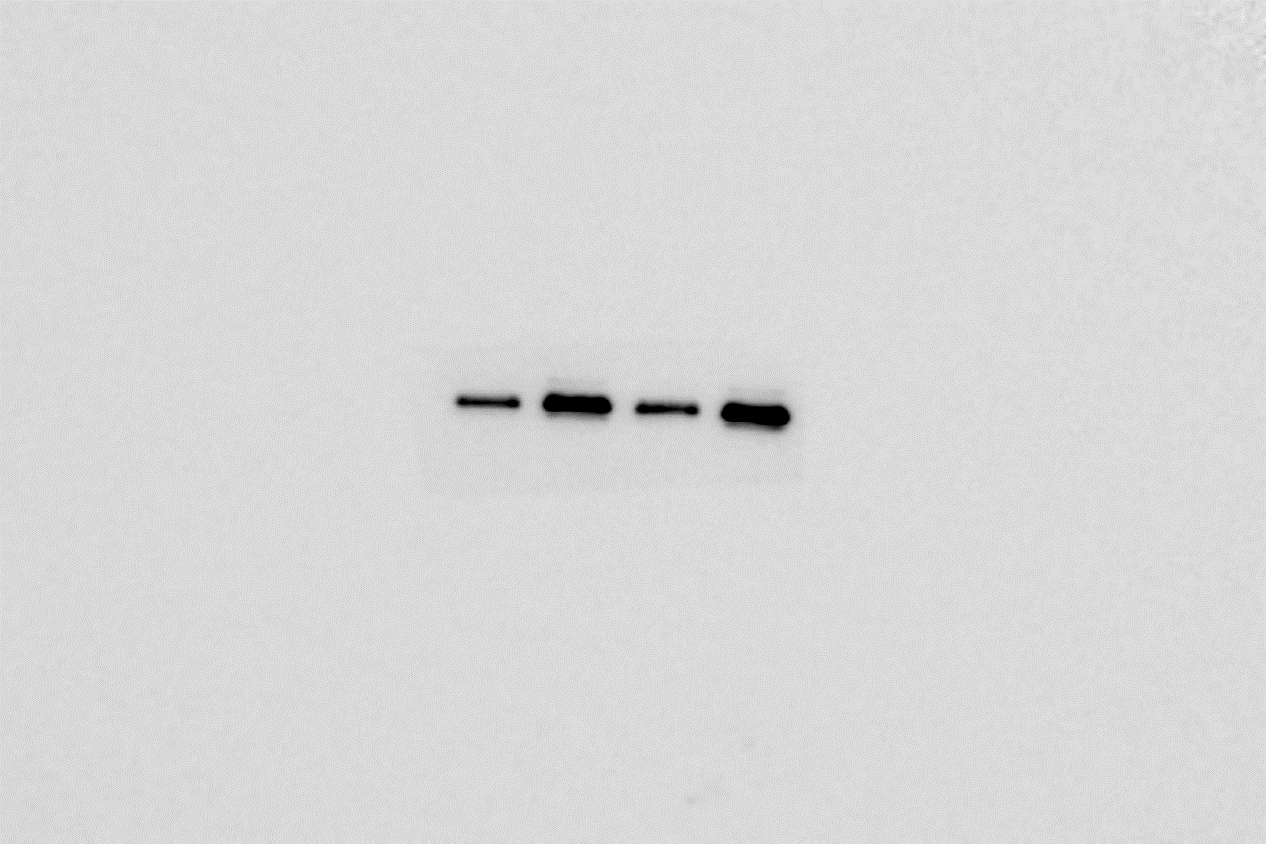


β-actin
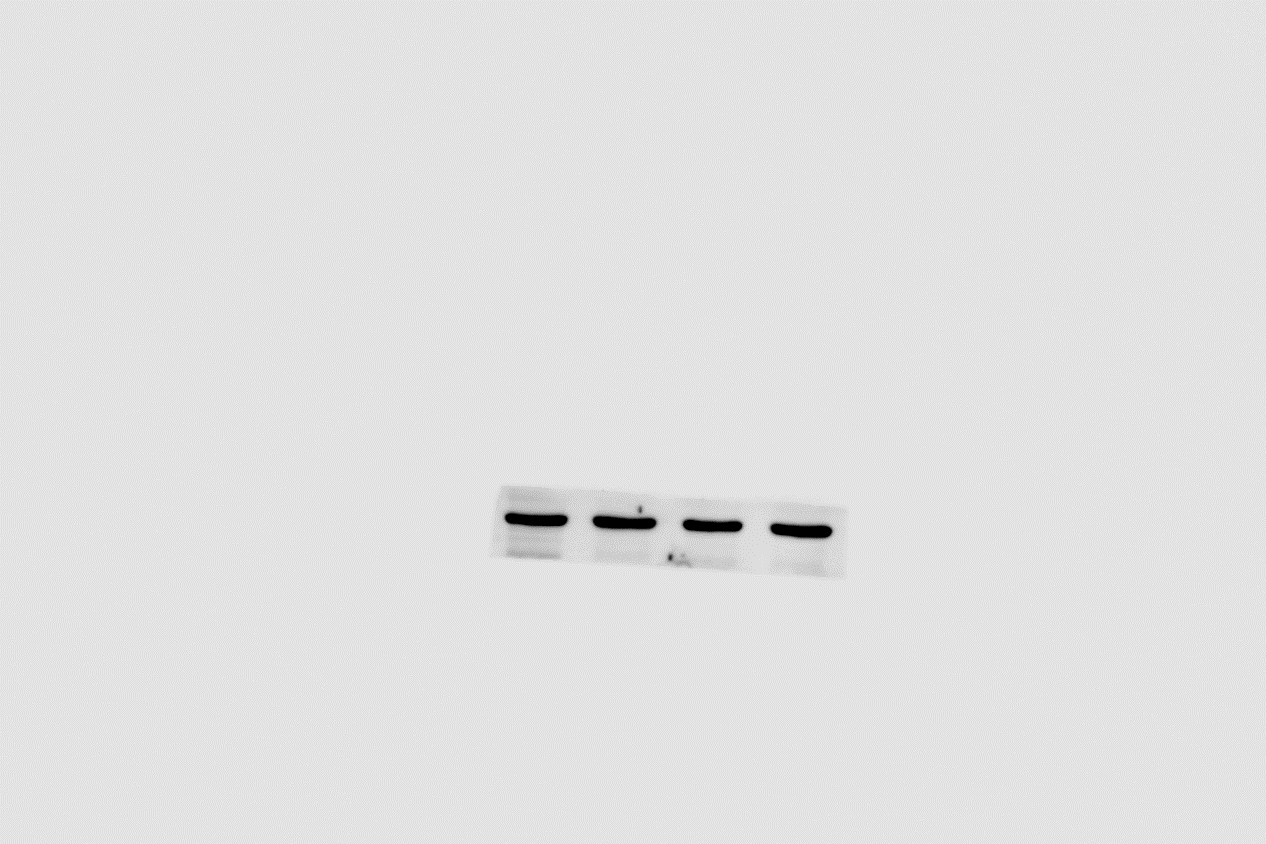


D NF-κB modulated the protein expression of NOX2 in TNF-α-stimulated A549 cells.

NOX2


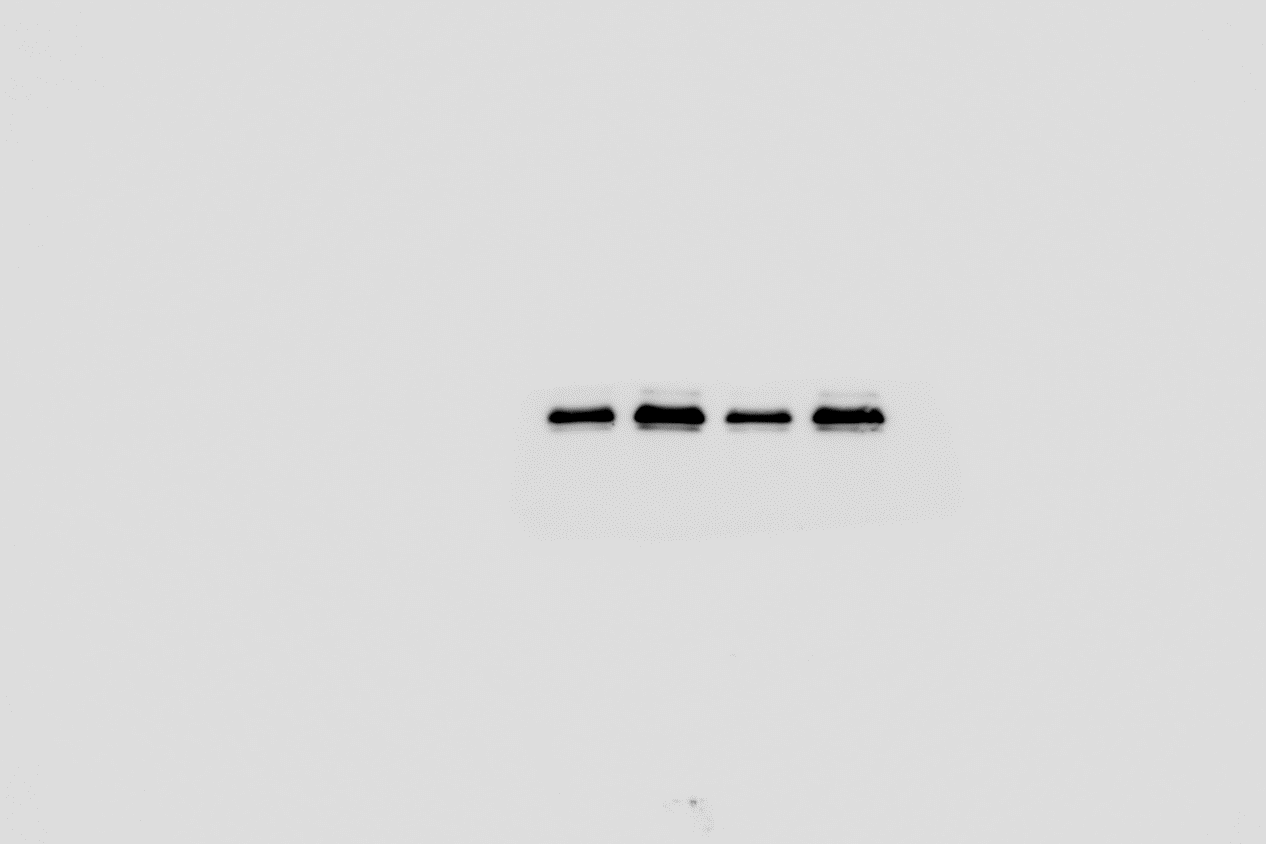


β-actin(（The right gel）


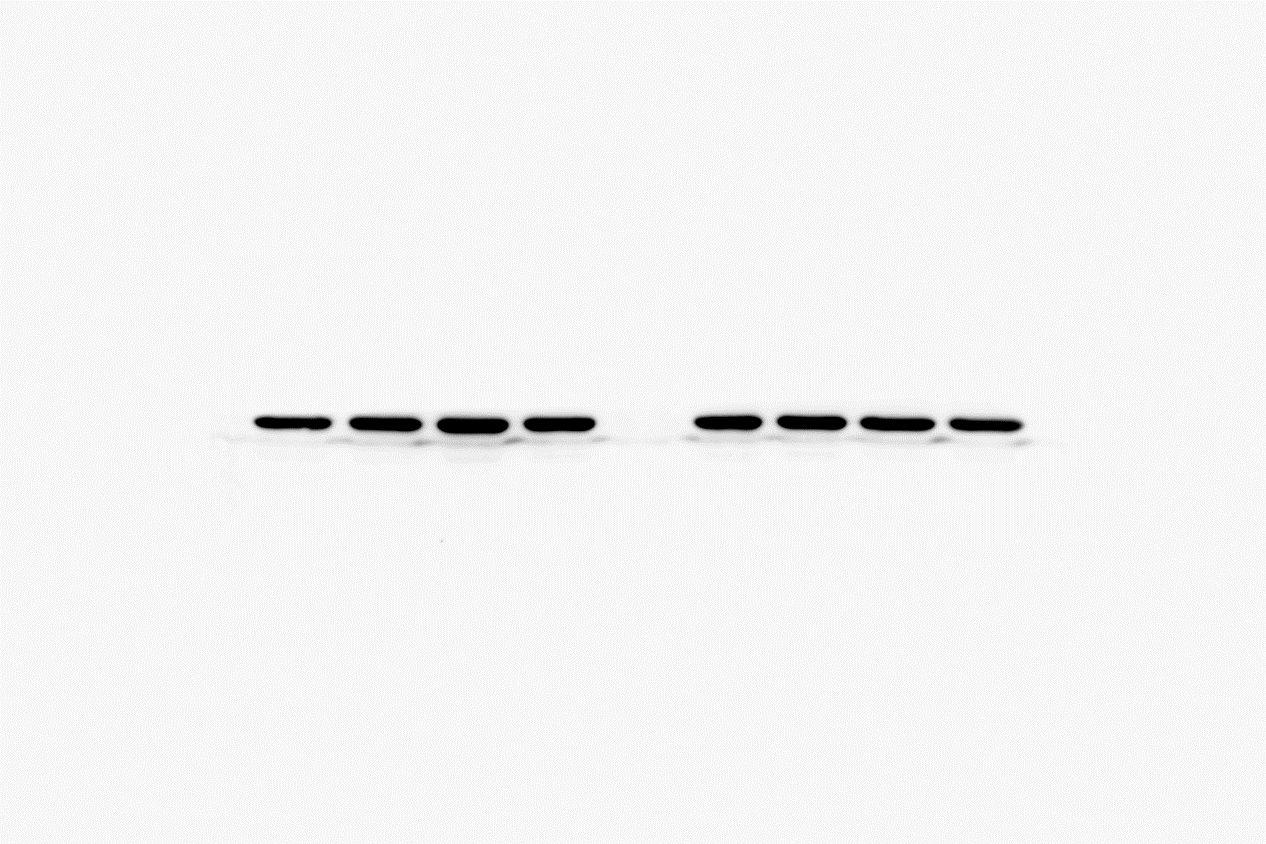


E NF-κB modulated the protein expression of NOX4 in TNF-α-stimulated A549 cells.

NOX4


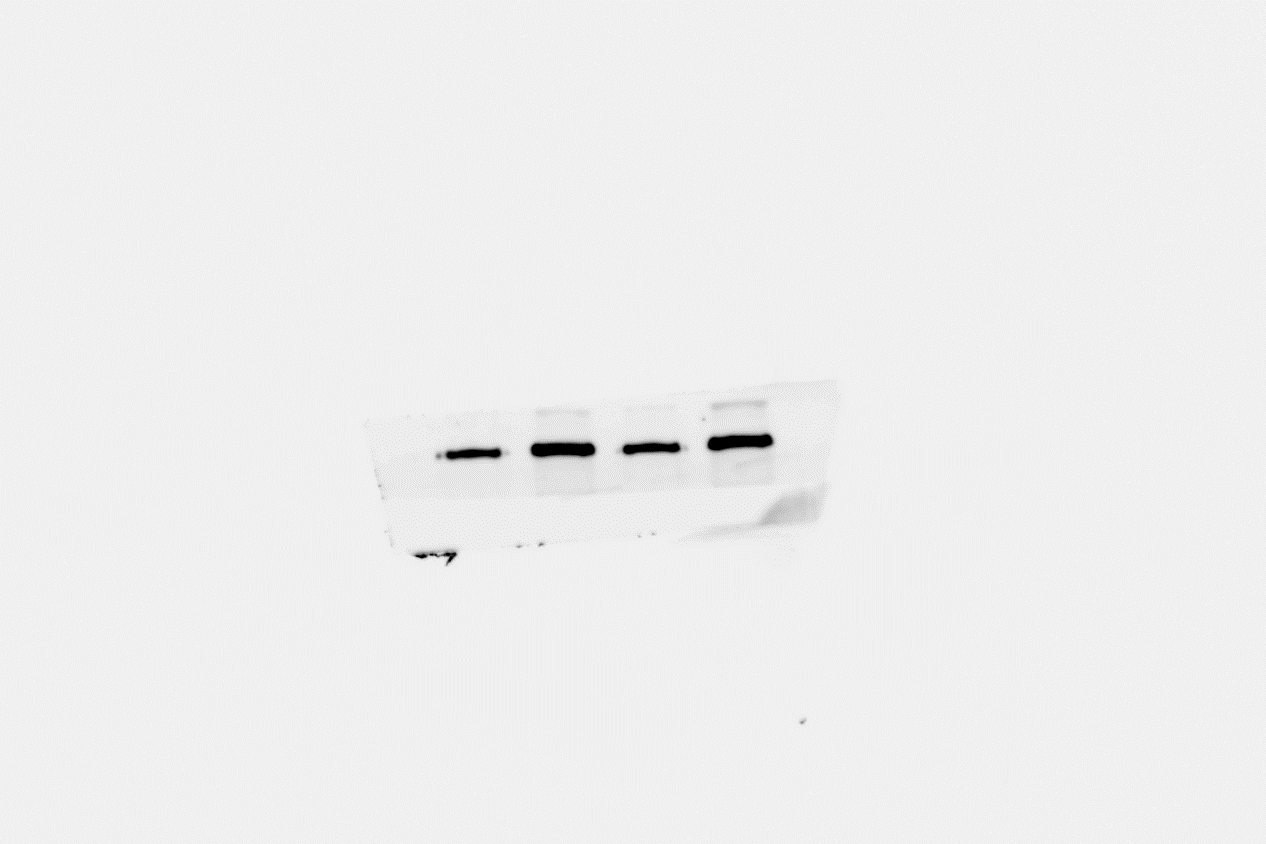


β-actin (The right gel)


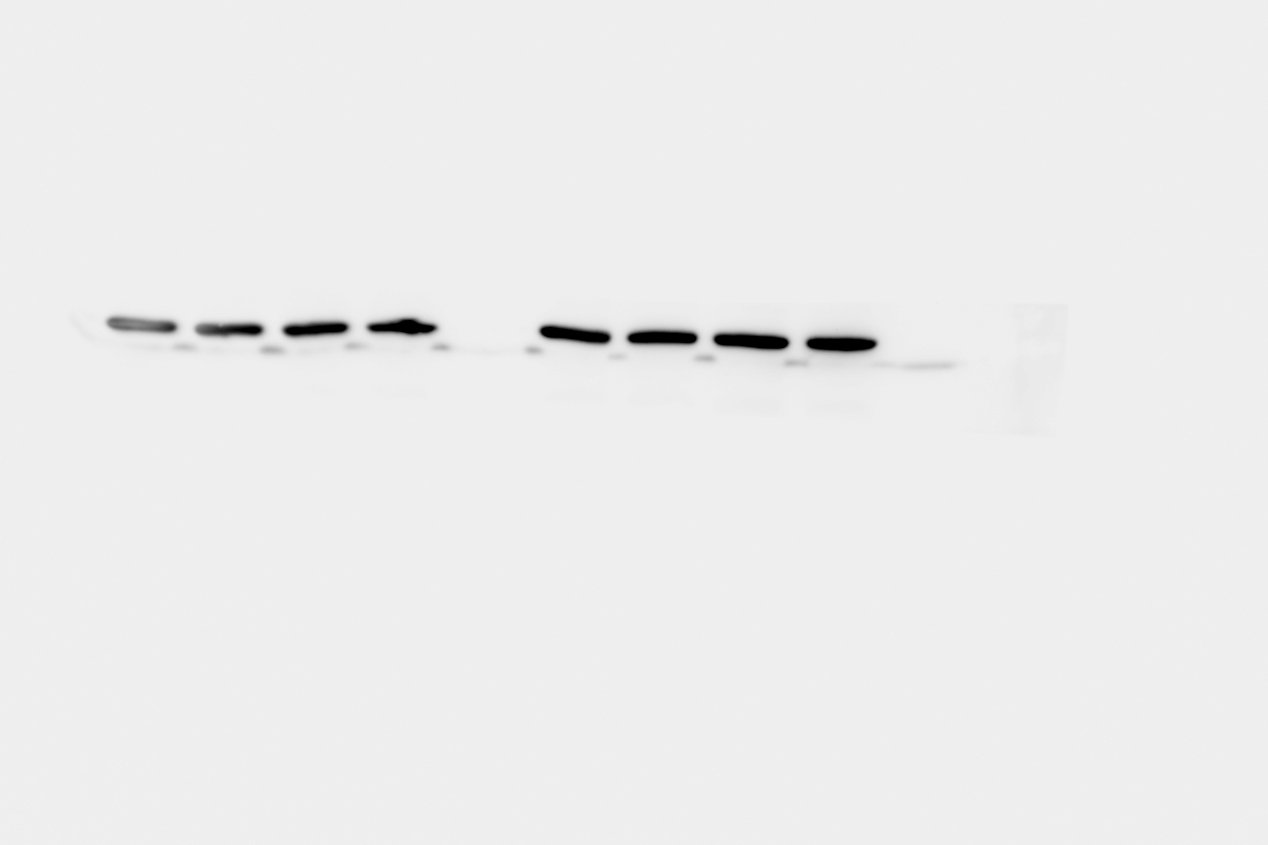

Supplement: Supplementary file 2 — Additional file 2. Supplementary Table 1. The concentration of ROS and MDA of different groups. Supplementary Table 2. The concentration of TAOS, SOD and TGSH of different groups. [file 12890_2021_1464_MOESM2_ESM.doc]
